# First report on tick-borne pathogens detected in ticks infesting stray dogs near butcher shops

**DOI:** 10.3389/fvets.2023.1246871

**Published:** 2023-09-18

**Authors:** Abid Ali, Shafi Ullah, Muhammad Numan, Mashal M. Almutairi, Abdulaziz Alouffi, Tetsuya Tanaka

**Affiliations:** ^1^Department of Zoology, Abdul Wali Khan University Mardan, Mardan, Pakistan; ^2^Department of Pharmacology and Toxicology, College of Pharmacy, King Saud University, Riyadh, Saudi Arabia; ^3^King Abdulaziz City for Science and Technology, Riyadh, Saudi Arabia; ^4^Laboratory of Infectious Diseases, Joint Faculty of Veterinary Medicine, Kagoshima University, Kagoshima, Japan

**Keywords:** ticks, pathogens, stray dogs, butcher, one-health

## Abstract

Public health is a major concern for several developing countries due to infectious agents transmitted by hematophagous arthropods such as ticks. Health risks due to infectious agents transmitted by ticks infesting butcher-associated stray dogs (BASDs) in urban and peri-urban regions have been neglected in several developing countries. To the best of the authors’ knowledge, this is the first study assessing public health risks due to ticks infesting BASDs in Pakistan’s urban and peri-urban areas. A total of 575 ticks (390 from symptomatic and 183 from asymptomatic BASDs) were collected from 117 BASDs (63 symptomatic and 54 asymptomatic); the ticks belonged to 4 hard tick species. A subset of each tick species’ extracted DNA was subjected to polymerase chain reaction (PCR) to amplify the 16S rDNA and *cox1* sequences of the reported tick species, as well as bacterial and protozoal agents. The ticks’ 16S rDNA and *cox1* sequences showed 99–100% identities, and they were clustered with the sequence of corresponding species from Pakistan and other countries in phylogenetic trees. Among the screened 271 ticks’ DNA samples, *Anaplasma* spp. was detected in 54/271 (19.92%) samples, followed by *Ehrlichia* spp. (*n* = 40/271, 14.76%), *Rickettsia* spp. (*n* = 33/271, 12.17%), *Coxiella* spp. (*n* = 23/271, 4.48%), and *Hepatozoon canis* (*n* = 9/271, 3.32%). The obtained sequences and phylogenetic analyzes revealed that the pathogens detected in ticks were *Ehrlichia minasensis*, *Ehrlichia* sp., *Hepatozoon canis*, *Coxiella burnetii*, *Coxiella* sp., *Anaplasma capra*, *Anaplasma platys*, *Anaplasma* sp., *Rickettsia massiliae*, “*Candidatus* Rickettsia shennongii” and *Rickettsia aeschlimannii*. Tick-borne pathogens such as *E. minasensis*, *H. canis*, *A. capra*, *A. platys*, and *R. aeschlimannii*, were detected based on the DNA for the first time in Pakistan. This is the first report on public health risks due to ticks infesting BASDs. These results not only provided insights into the occurrence of novel tick-borne pathogens in the region but also revealed initial evidence of zoonotic threats to both public health and domestic life.

## Introduction

The availability of survival resources (i.e., shelter, food, and water) affects the dog population in a region and directly correlates with the human population (1). Stray dogs are highly vulnerable to vector-borne diseases (VBDs) of zoonotic concern and, consequently, pose threats to human and animal health (2). Although the exact number globally is unknown, 75% out of 700 million dogs are considered stray dogs (3). They are the first farmed animals and are closely associated with humans on cultural, social, and economic levels (4). Pakistan is a developing country, and contemporary social issues have imposed an epidemic of stray dogs that may affect both the welfare of the animals and the public’s health, especially in urban and peri-urban areas (5). The exact number of these free-roaming stray dogs in the country is unknown, and the estimated stray dog population may have reached up to 3 million (6).

Tick species infesting stray dogs in the urban and peri-urban areas include *Rhipicephalus sanguineus* senso lato (s.l.), *Haemaphysalis elliptica*, *Rhipicephalus microplus*, *Haemaphysalis erinacei*, and *Haemaphysalis parva* (7, 8). Knowledge of tick identification is vital for surveillance to minimize the zoonotic consequences associated with tick-borne pathogens (9). Despite the morphological identification, genetic characterization using various genetic markers such as 16S rDNA and *cox1* is a suitable approach to determine the taxonomic status of a tick species (10–16).

Stray dogs being important reservoirs for tick-borne zoonotic agents may pose a threat to public health by causing anaplasmosis, rickettsiosis, babesiosis, query fever (Q fever), and ehrlichiosis (17–19). The genus *Ehrlichia* has seven determined species: *Ehrlichia canis*, *Ehrlichia chaffeensis*, *Ehrlichia ruminantium*, *Ehrlichia muris*, *Ehrlichia ewingii*, *Ehrlichia ovina*, and *Ehrlichia minasensis* (20, 21). Ticks responsible for the transmission of *Ehrlichia* spp. include species of the genera *Rhipicephalus*, *Hyalomma*, *Dermacentor*, *Amblyomma*, and *Ixodes* (20, 22). Canine monocytic ehrlichiosis (CME) is the most common tick-borne zoonotic disease that affects dogs globally (23). The brown dog tick, *Rh. sanguineus*, primarily vectors the etiological agent of CME (23). *Hepatozoon canis* causes canine hepatozoonosis, which is widely distributed in South America, Africa, Asia, and South Europe (24). Q fever is a tick-borne infection caused by *Coxiella burnetii* (25). Tick species of the genera *Rhipicephalus*, *Hyalomma*, *Amblyomma*, *Dermacentor*, and *Ixodes* transmit this pathogen (26). Anaplasmataceae, consisting of different genera, such as *Anaplasma*, *Ehrlichia*, *Neorickettsia*, and *Wolbachia*, contains vector-borne bacteria that are mainly transmitted by ticks (27). Tick species belonging to the genera *Amblyomma*, *Dermacentor*, *Haemaphysalis*, *Ixodes*, and *Rhipicephalus* transmit *Anaplasma* spp. to various hosts, including dogs (8, 28). *Anaplasma platys* and *Anaplasma phagocytophilum* are the most commonly detected species in dogs responsible for zoonotic diseases (2). Dogs are reservoir hosts for various spotted fever group agents (SFG) transmitted by *Rh. sanguineus* ticks (20). Rickettsial infections transmitted by *Rh. sanguineus* include the spotted fever group rickettsiosis caused by *R. massiliae* (20, 29), Mediterranean spotted fever (MSF) caused by *Rickettsia conorii* (30), and Rocky Mountain spotted fever (RMSF) caused by *Rickettsia rickettsii* (23). Dogs and their associated ticks play a major role in the ecology and epidemiology of several *Rickettsia* species, including *Rickettsia aeschlimannii* (23, 30).

The percentage of stray or semi-domesticated dogs is inversely correlated with the gross domestic product (GDP) *per capita*. Additionally, the burden of canine zoonotic parasites has been correlated with poverty, but in some cases, accurate and updated estimates regarding this data are lacking (31). Raising community knowledge, raising awareness of unhealthy practices, and ensuring preventive measures against disease-causing agents are crucial for effectively controlling ticks and tick-borne pathogens (32). Stray dogs survive through edible wastes from humans, especially from butchered surroundings in urban and peri-urban areas, mostly in developing countries. An overabundance of butcher trash appears to result from the lack of proper waste management and disposal, mostly in low-income countries (33). The abundance of discarded meat trash that is readily available for feed mostly results in the presence of large numbers of butcher-associated stray dogs (BASDs) close to butcher shops. Pakistan is a low and middle-income country (LMIC) that is heavily populated and socioeconomically underdeveloped (6, 11, 29). Due to the lack of veterinary supervision and management of butcher waste, it attracts hungry stray dogs who feed upon it. In Pakistan, butcher shops on road and street sides are major reasons for the spread of ticks that infest BASDs. No previous study has assessed public health risks and the epidemiological status of ticks and tick-borne pathogens on BASDs. This study aimed to identify the ticks infesting BASDs and to molecularly detect associated tick-borne pathogens that pose public health threats in urban and peri-urban areas.

## Materials and methods

### Study model

**Figure 1:**
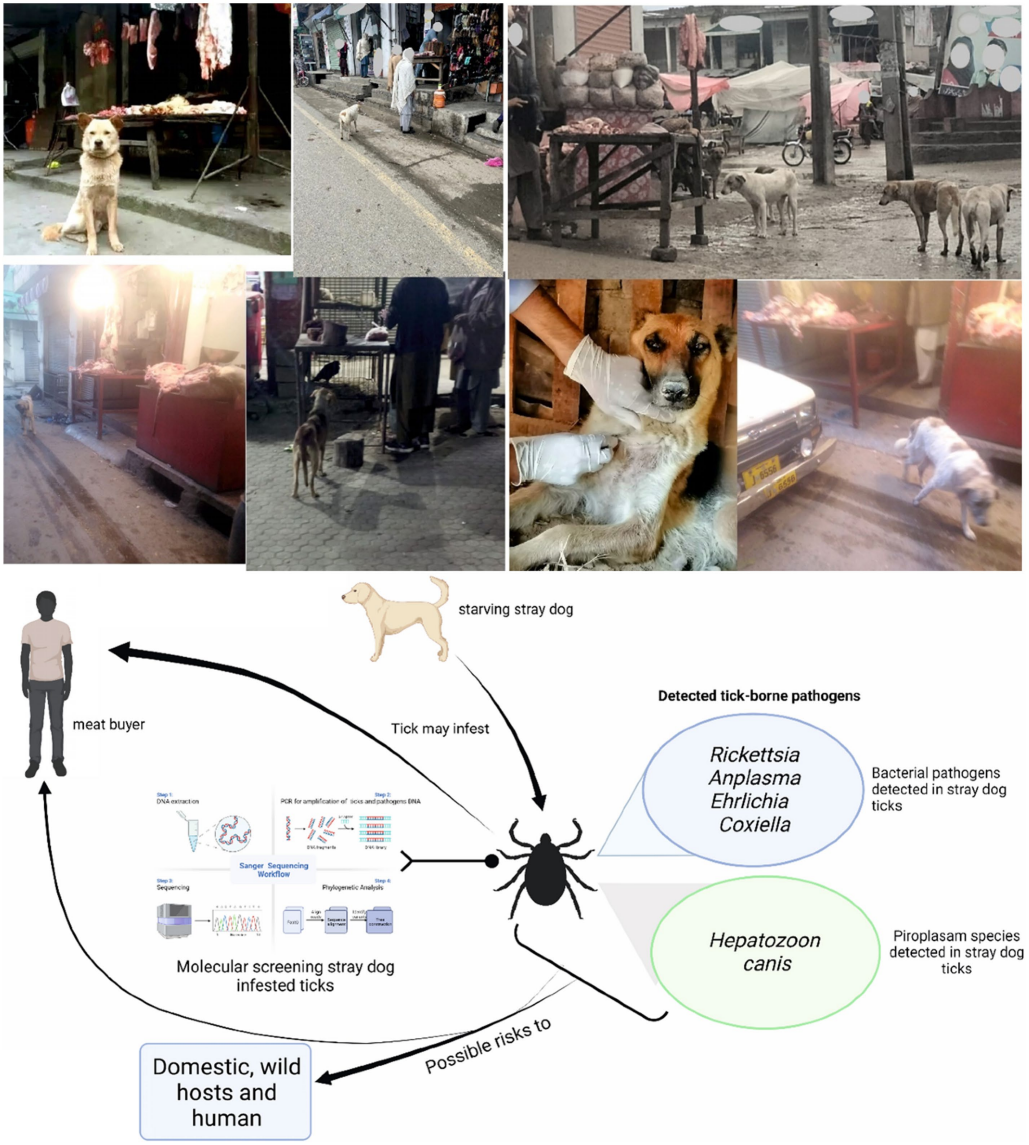
Study model describing the possible risks related to butcher-associated stray dogs. Parts of the figures were drawn by using pictures from Servier Medical Art (http://smart.servier.com/), licensed under a Creative Commons Attribution 3.0 Unported License (accessed 02 May 2023).

This study was designed to detect infectious agents harbored by ticks infesting BASDs. Stray dogs were observed in or near butcher shops. To test the hypothesis that meat buyers in the study area might be at risk due to tick-borne pathogens, ticks were observed on BASDs mostly in or near butcher shops. Ticks were collected from these BASDs and subjected to molecular detection for various bacterial and protozoal pathogens (Figure 1).

### Study area

**Figure 2:**
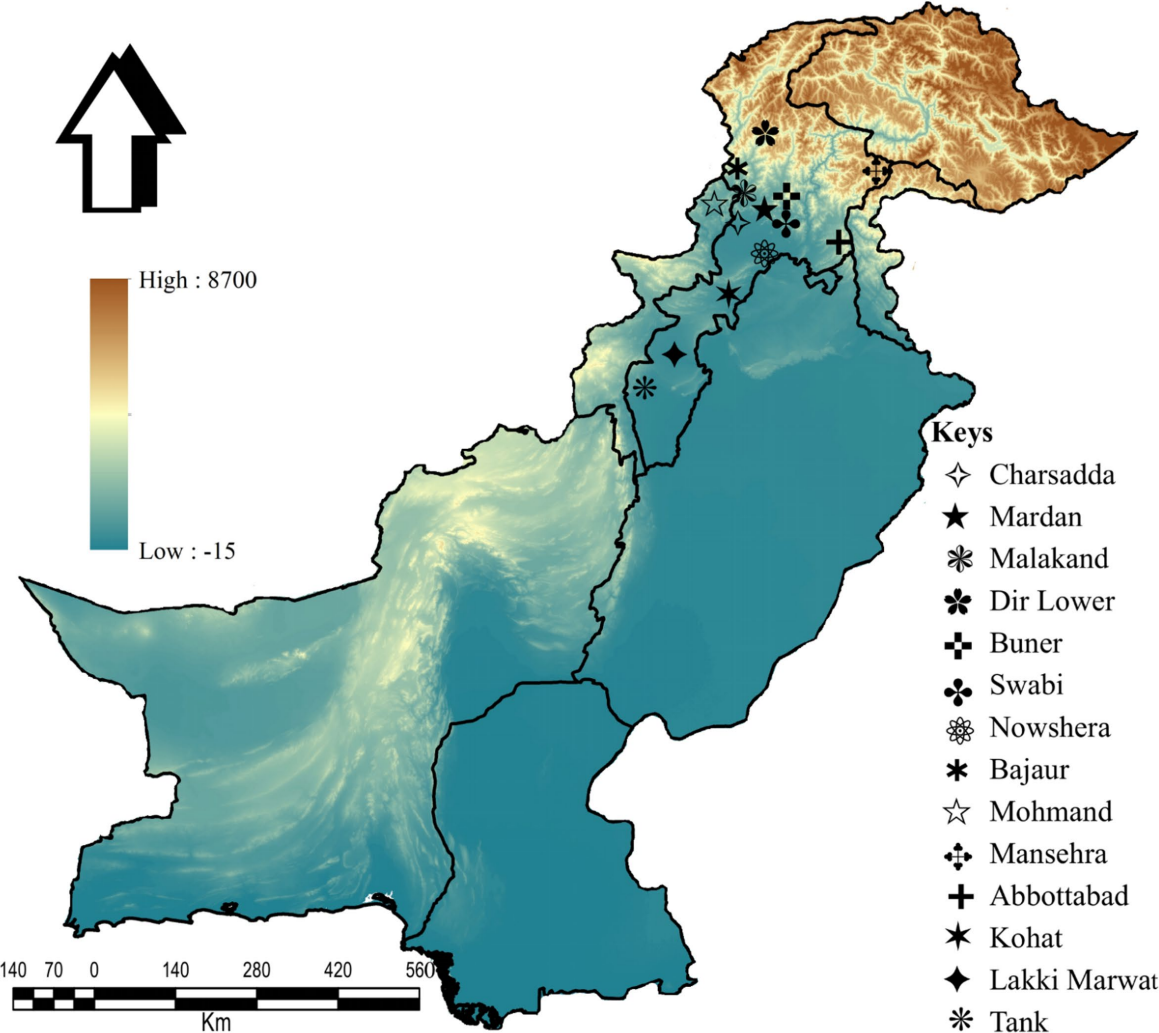
Map showing the collection sites of tick samples in the selected districts.

This study was conducted in 14 districts: Charsadda (34°09′46.2”N, 71°45′08.2″E), Mardan (34°11′41.8”N, 72°03′03.3″E), Malakand (34°33′31.4”N, 71°56′50.1″E), Lower Dir (34°53′20.1”N, 71°53′15.2″E), Buner (34°28′48.9”N, 72°31′13.5″E), Swabi (34°07′44.0”N, 72°27′23.9″E), Nowshera (34°00′12.4”N, 71°59′42.1″E), Bajaur (34°45′55.6”N, 71°30′54.0″E), Mohmand (34°29′05.3”N, 71°21′41.3″E), Mansehra (34°19′44.4”N, 73°12′15.3″E), Abbottabad (34°10′39.4”N, 73°14′09.4″E), Kohat (33°33′46.2”N, 71°28′20.4″E), Lakki Marwat (32°36′12.4”N, 70°54′22.1″E), and Tank (32°12′52.5”N, 70°23′09.8″E) in KP, Pakistan. The Global Positioning System (GPS) was used for the geographical coordinates of the collection sites, and the study map was designed using ArcGIS v. 10.3.1 (ESRI, Redlands, CA, United States; Figure 2).

### Tick collection and preservation

Ticks were collected from BASDs in or near butcher shops from June 2021 to May 2022 in 14 selected districts. They were carefully isolated from the host body using tweezers to avoid external damage to the specimens. The specimens were rinsed in distilled water, followed by 70% ethanol, and preserved in 100% ethanol.

The examined BASDs consisted of both symptomatic and asymptomatic dogs. Symptomatic dogs were characterized by accompanying symptoms such as diarrhea, vomiting, limb swelling, rashes, itching, seizures, fatigue, scabs or skin lesions, and hair loss.

### Morphology of ticks

The collected tick specimens were identified morphologically at the species level under a stereomicroscope (SZ61, Olympus Corporation, Tokyo, Japan) using standard morphological identification keys (29, 34).

### DNA extraction and PCR

A total of 271 ticks (193 and 78 from symptomatic and asymptomatic BASDs, respectively) were randomly selected for molecular analyzes. Before DNA extraction, the specimens were washed in distilled water, followed by absolute ethanol. Then, they were kept in an incubator (30–45 min) until dry (20–30 min). Tick specimens were cut with sterile scissors and homogenized in phosphate-buffered saline using a micro pestle, and DNA was extracted by employing the phenol-chloroform method (35). A NanoDrop (Nano-Q, Optizen, Daejeon, South Korea) was used to quantify the DNA, and the DNA samples were stored at −20°C for further examination.

The extracted DNA was subjected to a polymerase chain reaction (PCR; GE-96G, BIOER, Hangzhou, China) to amplify mitochondrial 16S ribosomal DNA (16S rDNA) and cytochrome C oxidase 1 (*cox1*) fragments for tick identification. Each PCR reaction mixture contained 25 μL volume, comprising 1 μL each primer (forward and reverse; 10 μM), 8.5 μL PCR water “nuclease-free,” 12.5 μL Dream*Taq* green MasterMix (2X; Thermo Scientific, Waltham, MA, United States), and 2 μL of template DNA (100 ng/μl).

The extracted DNA was utilized for screening various tick-borne pathogens based on different partial genes, such as 16S rDNA (*Anaplasma*/*Ehrlichia* spp.), *groEL* (*Coxiella* spp.), *dsb* (*Ehrlichia* spp.), 18S rDNA (*Hepatozoon* spp.), and *gltA, ompA*, and *ompB* (*Rickettsia* spp.). The nested PCR was used in the case of the *groEL* partial gene. Each PCR experiment contained a negative control (PCR water instead of the template DNA) and a positive control (the DNA of *Rh. microplus* for ticks, *R. massiliae* for bacterial species, and *Theileria annulata* for protozoal species). The primers used in the current study and their thermocycler conditions are given in Supplementary Table S1.

The PCR products were electrophoresed on 1.5% agarose gel, and the amplified samples were visualized under ultraviolet (UV) light using a gel documentation system (BioDoc-IT™ Imaging Systems UVP, LLC, Upland, United States). The PCR products that showed the expected size results were purified using a commercial PCR clean-up kit (Macherey-Nagel, Duren, Germany) following the manufacturer’s instructions.

### DNA sequencing and phylogenetic analysis

PCR products of 16S rDNA and *cox1* for ticks, as well as 16S rDNA, *groEL*, *dsb*, 18S rDNA, *gltA*, *ompA*, and *ompB* for associated tick-borne pathogens, were sent for capillary bidirectional sequencing (Macrogen Inc., Seoul, South Korea). The obtained sequences were trimmed and assembled using SeqMan v. 5 (DNASTAR, Inc., Madison/WI, United States) to remove poor reading sequences. The obtained multiple identical sequences for each gene were considered a consensus sequence and subjected to the basic local alignment search tool (BLAST) at the National Center for Biotechnology Information (NCBI). Sequences with maximum identities were downloaded in FASTA format from the NCBI. The obtained sequences were aligned with the downloaded sequences using ClustalW multiple alignments in BioEdit Sequence Alignment Editor v. 7.0.5 (36). The coding nucleotide sequences were aligned using MUSCLE (37). Phylogenetic trees were constructed individually for each partial sequence of tick and associated tick-borne pathogens, by employing the maximum likelihood (ML) method with Kimura 2-parameter in Molecular Evolutionary Genetics Analysis (MEGA-X), accompanied by a bootstrapping value of 1,000 (38).

### Statistical analyzes

Tick data and associated pathogen information from all 14 districts were subjected to descriptive statistical analysis, and graphs were designed in Microsoft Excel v. 2016 (Microsoft Office 365®). The minimum infection rate (MIR) was used to estimate the prevalence of infected ticks. To accomplish this, the number of PCR product ticks was divided by the total number of collected ticks (39).

## Results

A survey in the selected districts resulted in an overall collection of 575 ticks (392 and 183 from symptomatic and asymptomatic BASDs, respectively) from 117 BASDs (63 symptomatic and 54 asymptomatic), comprising four hard tick species. Among the identified ticks, *Rh. sanguineus* was the most prevalent tick species (*n* = 292/575, 50.78%), followed by *Rh. haemaphysaloides* (*n* = 127/575, 22.08%), *Rh. turanicus* counting (*n* = 87/575, 15.13%), and *Rh. microplus* clade C (*n* = 69/575, 12.00%). The spatial distribution of the collected ticks revealed that Mardan, Lakki Marwat, and Charsadda had the highest tick counts of 58, 53, and 51 ticks, respectively. The Malakand district (27) had the least number of collected ticks among the selected districts.

Of the 575 tick specimens, 271 (47.13%; 193 and 78 from symptomatic and asymptomatic BASDs, respectively) were subjected to PCR for the molecular screening of various tick-borne pathogens, including *Ehrlichia*, *Anaplasma*, *Rickettsia*, *Coxiella* and *Hepatozoon* species (Supplementary Table S1). According to the PCR-based screening and subsequent sequencing of targeted bacterial and protozoal agents, *Anaplasma* spp. were the most prevalent (*n* = 54/271, 19.92%), followed by *Ehrlichia* spp. (*n* = 40/271, 14.76%), *Rickettsia* spp. (*n* = 33/271, 12.17%), *Coxiella* spp. (*n* = 23/271, 8.49%), and *Hepatozoon* spp. (*n* = 9/271, 3.32%) in the ticks infesting BASDs (Table 1). None of the ticks was found positive for multiple pathogens.

**Table 1:** Distribution pattern of butcher-associated stray dog ticks and the detection of bacterial and protozoal pathogens in these ticks.

Districts	Tick species	Number of hosts (symptomatic/asymptomatic)	Ticks collected (S/A)* Male, Female, Nymph	Total (S/A)*	Subjected to PCR (S/A)*	Bacterial species (S/A)*				Protozoan (S/A)*
						*Ehrlichia* spp.	*Anaplasma* spp.	*Rickettsia* spp.	*Coxiella* spp.	*Hepatozoon* spp.
Charsadda	*Rh. sanguineus*	1/2	6/2 M, 7/4F, 2/2 N	15/8	7/3	1/1	2/1	2/0	1/1	1/0
	*Rh. haemaphysaloides*	1/1	3/2 M, 3/3F, 1/1 N	7/6	4/3	1/1	1/1	0	1/0	0
	*Rh. turanicus*	1/1	1/1 M, 2/1F, 2/0 N	5/2	4/1	0	1/0	1/0	0	0
	*Rh. microplus*	1/1	1/0 M, 3/1F, 2/1 N	6/2	2/2	1/0	0	0	0	0
Total		4/5	11/5 M, 15/9F, 7/4 N	33/18	17/9	3/2	4/2	3/0	2/1	1/0
Mardan	*Rh. sanguineus*	3/1	6/6 M, 10/4F, 2/2 N	18/12	11/4	1/1	4/1	3/1	2/1	1/0
	*Rh. haemaphysaloides*	1/1	3/2 M, 4/3F, 1/1 N	8/6	3/2	0	1/1	1/0	1/1	0
	*Rh. turanicus*	1/1	2/2 M, 1/1F, 2/0 N	5/3	2/1	0	1/0	0	1/0	0
	*Rh. microplus*	1/1	2/1 M, 2/0F, 1/0 N	5/1	4/2	0	1/0	0	1/0	0
Total		6/4	13/11 M, 17/8F, 6/3 N	36/22	20/9	1/1	7/2	4/1	5/2	1/0
Malakand	*Rh. sanguineus*	1/1	3/2 M, 4/3F, 1/0 N	8/5	7/5	0	3/1	0	1/1	1/0
	*Rh. haemaphysaloides*	1/1	2/0 M, 2/1F, 1/0 N	5/1	5/1	2/0	1/0	1/0	1/0	0
	*Rh. turanicus*	1/1	1/0 M, 1/1F, 0 N	2/1	2/1	0	0	0	0	1/0
	*Rh. microplus*	1/1	1/0 M, 2/1F, 1/0 N	4/1	4/1	0	1/0	1/0	1/0	1/0
Total		4/4	7/2 M, 9/6F, 3/0 N	19/8	18/8	2/0	5/1	2/0	3/1	3/0
Dir Lower	*Rh. sanguineus*	1/1	4/4 M, 4/2F, 1/0 N	9/6	4/2	0	1/0	1/1	1/0	1/0
	*Rh. haemaphysaloides*	2/1	3/1 M, 3/2F, 1/1 N	7/4	4/2	2/0	0	1/0	1/0	0
	*Rh. turanicus*	1/1	1/0 M, 3/3F, 2/0 N	6/3	6/3	1/1	1/0	0	0	0
	*Rh. microplus*	1/1	1/1 M, 2/2F, 2/0 N	5/3	5/3	1/1	0	0	0	0
Total		5/4	9/6 M, 12/9F, 6/1 N	27/16	19/10	4/2	2/0	2/1	2/0	1/0
Buner	*Rh. sanguineus*	2/1	5/2 M, 7/2F, 2/2 N	14/6	3/1	1/0	1/0	1/0	0	0
	*Rh. haemaphysaloides*	1/1	2/1 M, 3/1F, 2/0 N	7/2	3/2	0	0	1/0	0	0
	*Rh. turanicus*	1/0	2/0 M, 3/0F, 0 N	5/0	4/0	0	0	1/0	0	0
	*Rh. microplus*	1/0	1/0 M, 1/0F, 1/0 N	3/0	3/0	0	0	0	0	0
Total		5/2	10/3 M, 14/3F, 5/2 N	29/8	13/3	1/0	1/0	3/0	0	0
Swabi	*Rh. sanguineus*	1/2	4/3 M, 3/3F, 3/2 N	10/8	3/1	0	1/1	1/0	0	0
	*Rh. haemaphysaloides*	1/1	2/1 M, 2/1F, 1/0 N	5/2	4/1	1/0	1/0	0	0	0
	*Rh. turanicus*	1/1	2/0 M, 2/1F, 0 N	4/1	2/0	0	1/0	0	0	0
	*Rh. microplus*	1/1	1/0 M, 1/1F, 0 N	2/1	2/1	0	0	0	1/0	0
Total		4/5	9/4 M, 8/6F, 4/2 N	21/12	11/3	1/0	3/1	1/0	1/0	0
Nowshera	*Rh. sanguineus*	1/1	4/4 M, 4/1F, 3/1 N	11/6	5/3	1/0	2/1	1/0	0	1/0
	*Rh. haemaphysaloides*	1/1	2/0 M, 3/3F, 0 N	5/3	4/2	0	1/0	0	0	1/0
	*Rh. turanicus*	1/1	1/0 M, 2/0F, 1/1 N	4/1	4/1	1/0	1/0	1/0	1/0	0
	*Rh. microplus*	1/1	1/0 M, 2/1F, 1/0 N	4/1	4/1	0	0	1/0	0	0
Total		4/4	8/4 M, 11/5F, 5/2 N	24/11	17/7	2/0	4/1	3/0	1/0	2/0
Bajaur	*Rh. sanguineus*	1/2	5/4 M, 6/5F, 3/3 N	14/12	2/1	0	1/1	0	0	1/0
	*Rh. haemaphysaloides*	1/1	2/1 M, 3/2F, 1/0 N	6/3	3/1	1/0	1/0	1/0	0	0
	*Rh. turanicus*	1/1	0 M, 2/1F, 1/0 N	3/1	2/1	2/0	0	0	0	0
	*Rh. microplus*	1/0	3/0 M, 1/0F, 1/0 N	5/0	1/0	0	0	0	0	0
Total		4/4	10/5 M, 12/8F, 6/3 N	28/16	8/3	3/0	2/1	1/0	0	1/0
Mohmand	*Rh. sanguineus*	2/1	5/4 M, 4/2F, 2/0 N	11/6	6/3	2/1	2/1	1/0	1/0	0
	*Rh. haemaphysaloides*	1/1	2/1 M, 2/0F, 1/1 N	5/2	2/1	0	1/0	1/0	0	0
	*Rh. turanicus*	1/1	2/0 M, 3/1F, 2/0 N	7/1	3/1	0	0	0	0	0
	*Rh. microplus*	1/1	1/0 M, 3/1F, 0 N	4/1	2/0	0	0	0	0	0
Total		5/4	10/5 M, 12/4F, 5/1 N	27/10	13/5	2/1	3/1	2/0	1/0	0
Mansehra	*Rh. sanguineus*	1/1	8/4 M, 4/2F, 1/1 N	13/7	3/1	1/0	1/1	1/0	0	0
	*Rh. haemaphysaloides*	1/1	1/0 M, 2/2F, 2/0 N	5/2	2/1	0	1/0	1/0	0	0
	*Rh. turanicus*	1/1	2/0 M, 2/1F, 2/0 N	6/1	2/1	1/0	0	1/0	0	0
	*Rh. microplus*	1/1	0 M, 3/2F, 1/0 N	4/2	3/1	0	0	0	0	0
Total		4/4	11/4 M, 11/7F, 6/1 N	28/12	10/4	2/0	2/1	3/0	0	0
Abbottabad	*Rh. sanguineus*	2/1	7/4 M, 4/3F, 4/1 N	15/8	3/1	1/1	2/0	0	0	0
	*Rh. haemaphysaloides*	1/1	3/0 M, 2/2F, 1/0 N	6/2	2/1	1/0	0	0	0	0
	*Rh. turanicus*	1/1	1/0 M, 2/1F, 1/0 N	4/1	4/1	0	1/0	1/0	0	0
	*Rh. microplus*	1/1	1/1 M, 3/1F, 1/0 N	5/2	2/1	0	0	1/0	0	0
Total		5/4	12/5 M, 11/7F, 7/1 N	30/13	11/4	2/1	3/0	2/0	0	0
Kohat	*Rh. sanguineus*	2/1	6/1 M, 6/2F, 3/0 N	15/3	5/1	0	1/1	1/0	0	0
	*Rh. haemaphysaloides*	1/1	2/0 M, 2/1F, 1/0 N	5/1	2/1	1/0	0	0	0	0
	*Rh. turanicus*	1/1	0 M, 4/1F, 1/0 N	5/1	2/1	2/1	0	0	0	0
	*Rh. microplus*	1/1	0 M, 2/1F, 1/0 N	3/1	3/1	1/0	1/0	0	0	0
Total		5/4	8/1 M, 14/5F, 6/0 N	28/6	12/4	4/1	2/1	1/0	0	0
Lakki Marwat	*Rh. sanguineus*	1/1	6/6 M, 6/5F, 4/2 N	16/13	5/2	1/1	2/0	1/0	1/0	0
	*Rh. haemaphysaloides*	1/1	3/0 M, 5/2F, 2/0 N	10/2	2/1	0	1/0	0	1/0	0
	*Rh. turanicus*	1/1	1/0 M, 4/4F, 2/0 N	7/4	4/3	1/0	0	1/0	1/0	0
	*Rh. microplus*	1/0	0 M, 1/0F, 0 N	1/0	1/0	0	0	0	0	0
Total		4/3	10/6 M, 16/11F, 8/2 N	34/19	12/6	2/1	3/0	2/0	3/0	0
Tank	*Rh. sanguineus*	1/1	8/4 M, 4/3F, 4/0 N	16/7	4/1	1/0	1/0	1/0	1/0	0
	*Rh. haemaphysaloides*	1/1	2/2 M, 4/2F, 0 N	6/4	2/1	0	0	0	0	0
	*Rh. turanicus*	1/1	1/1 M, 1/0F, 1/0 N	3/1	3/1	1/0	1/0	1/0	0	0
	*Rh. microplus*	1/0	2/0 M, 1/0F, 0 N	3/0	3/0	0	0	0	0	0
Total		4/3	13/7 M, 10/5F, 5/0 N	28/12	12/3	2/0	2/0	2/0	1/0	0
Grand total		63/54 (117)	141/68 (209) M, 172/93 (265) F, 79/22 (101) N	392/183 (575)	193/78 (271)	31/9 (40)	43/11 (54)	31/2 (33)	19/4 (23)	9/0 (9)

*S, Symptomatic; A, Asymptomatic.

The results of the prevalence of symptomatic and asymptomatic BASDs in ticks, as well as the molecular screening of tick-associated bacterial and protozoal pathogens, are presented in Table 1.

### DNA sequences and phylogeny of ticks

**Figure 3:**
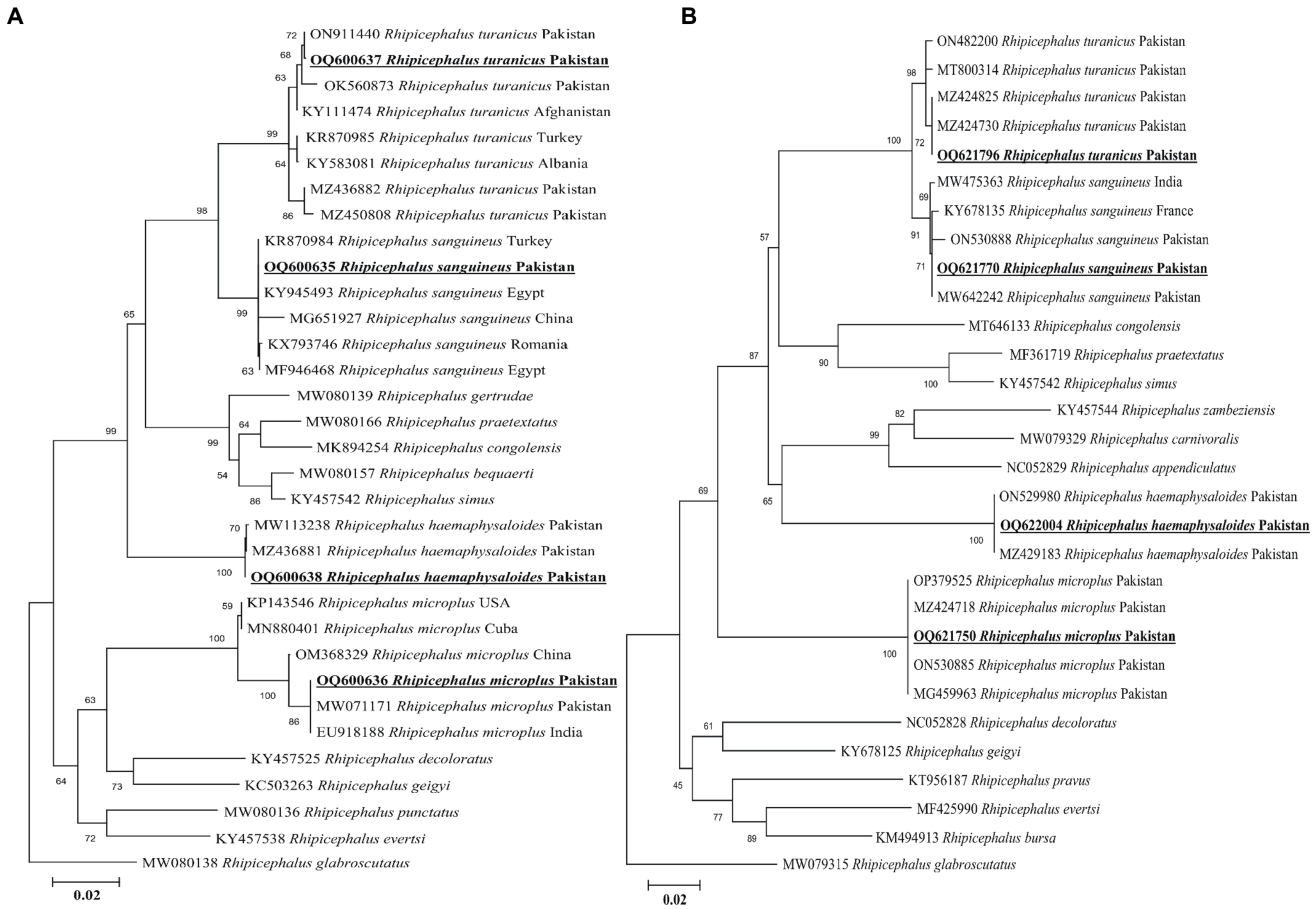
Maximum likelihood phylogenetic trees based on 16S rDNA **(A)** and *cox1* **(B)** partial sequences of *Rhipicephalus* spp. The *Rhipicephalus glabrosscutatus* sequence was used as an outgroup. The obtained sequences are shown in bold underline font.

Sequencing by BLAST analysis of the 16S rDNA for *Rh. sanguineus*, *Rh. turanicus*, *Rh. microplus*, and *Rh. haemaphysaloides* showed 99.24–100% maximum identities to the corresponding species. In the phylogenetic tree, the 16S rDNA sequences of *Rh. sanguineus*, *Rh. turanicus*, *Rhipicephalus microplus* (clade C), and *Rhipicephalus haemaphysaloides* were clustered with the corresponding species (Figure 3A).

According to the BLAST results, the obtained *cox1* sequences of *Rh. sanguineus*, *Rh. turanicus*, *Rh. microplus*, and *Rh. haemaphysaloides* shared 99.54–100% maximum identities with the corresponding species. In the phylogenetic tree, the *cox1* sequences of *Rh. sanguineus*, *Rh. turanicus*, *Rhipicephalus microplus*, and *Rhipicephalus haemephysalides* were clustered with the corresponding species (Figure 3B). All the obtained 16S rDNA and *cox1* sequences of *Rh. sanguineus*, *Rh. turanicus*, *Rh. microplus*, and *Rh. haemaphysaloides* were deposited to the GenBank (Supplementary Table S2).

### Prevalence of pathogens in ticks

**Figure 4:**
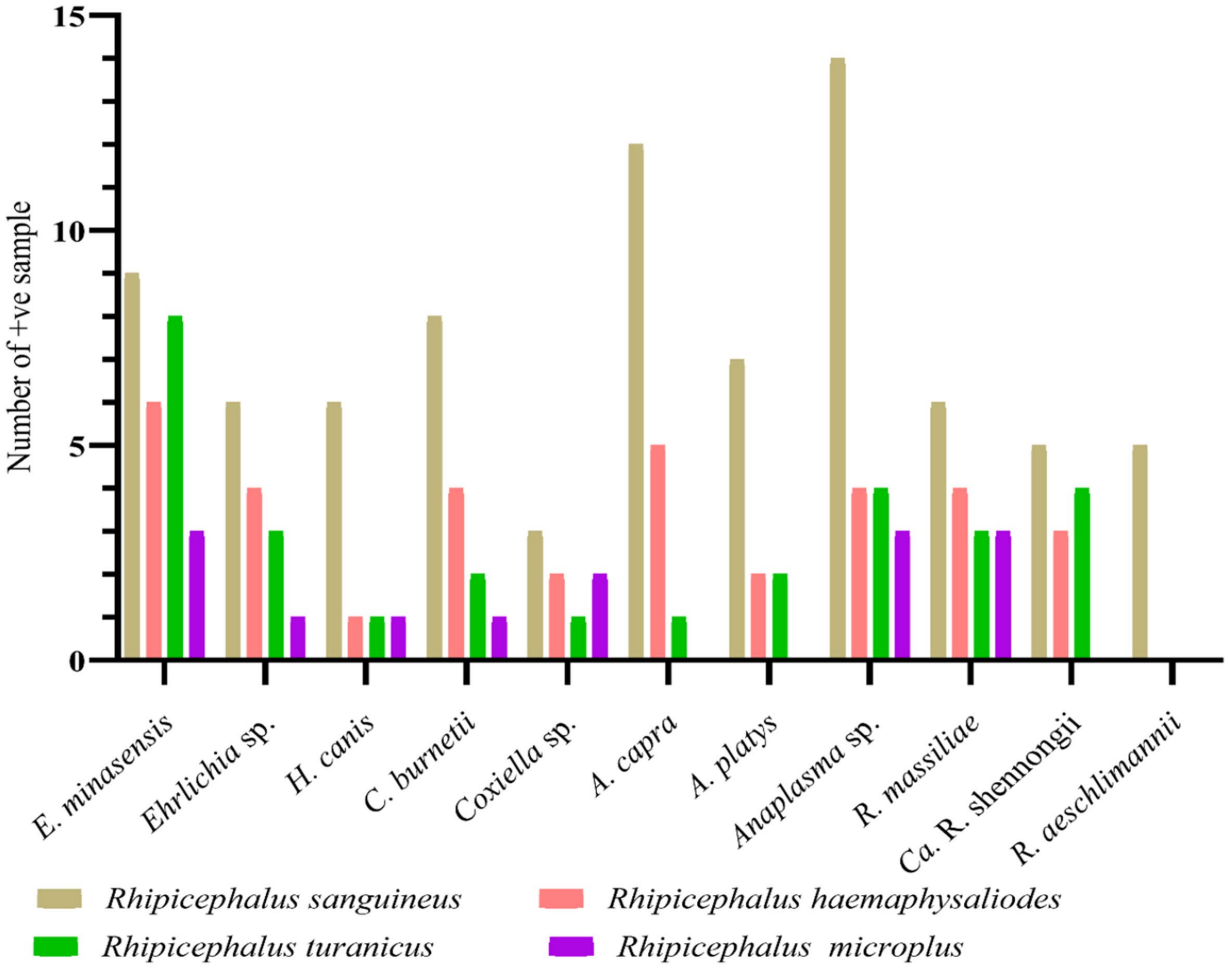
Molecular prevalence of detected pathogens in butcher-associated stray dog ticks.

Altogether, 271 out of 575 collected ticks were screened for detecting bacterial and protozoal pathogens. Among the four tested tick species, *Rh. sanguineus* was the most infected tick comprising 81 positive samples with a minimum infection rate of 29.88% (*n* = 81/271), followed by *Rh. haemaphysaloides* with a minimum infection rate of 12.91% (*n* = 35/271), *Rh. turanicus* with a minimum infection rate of 10.70% (*n* = 29/271), and *Rh. microplus* with a minimum infection rate of 5.16% (*n* = 14/271). All these minimum infection rates were statistically significant. Sequencing of the PCR-based amplified DNA resulted in the detection of eight pathogens–*E. minasensis*, *H. canis*, *C. burnetii*, *Anaplasma capra*, *A. platys*, *R. massiliae*, “*Candidatus* Rickettsia shennongii” and *R. aeschlimannii*. Three undetermined species, *Ehrlichia* sp., *Coxiella* sp., and *Anaplasma* sp. were also detected. All pathogens and undetermined species were detected in the collected four tested tick species, except *R. aeschlimannii*, which was detected only in *Rh. sanguineus*, whereas *A. capra*, *A. platys*, and “*Ca*. R. shennongii” were detected in *Rh. sanguineus*, *Rh. haemaphysaloides*, and *Rh. turanicus* (Figure 4).

### Sequence and phylogenetic analyzes of tick-borne pathogens

#### *Ehrlichia* spp.

**Figure 5:**
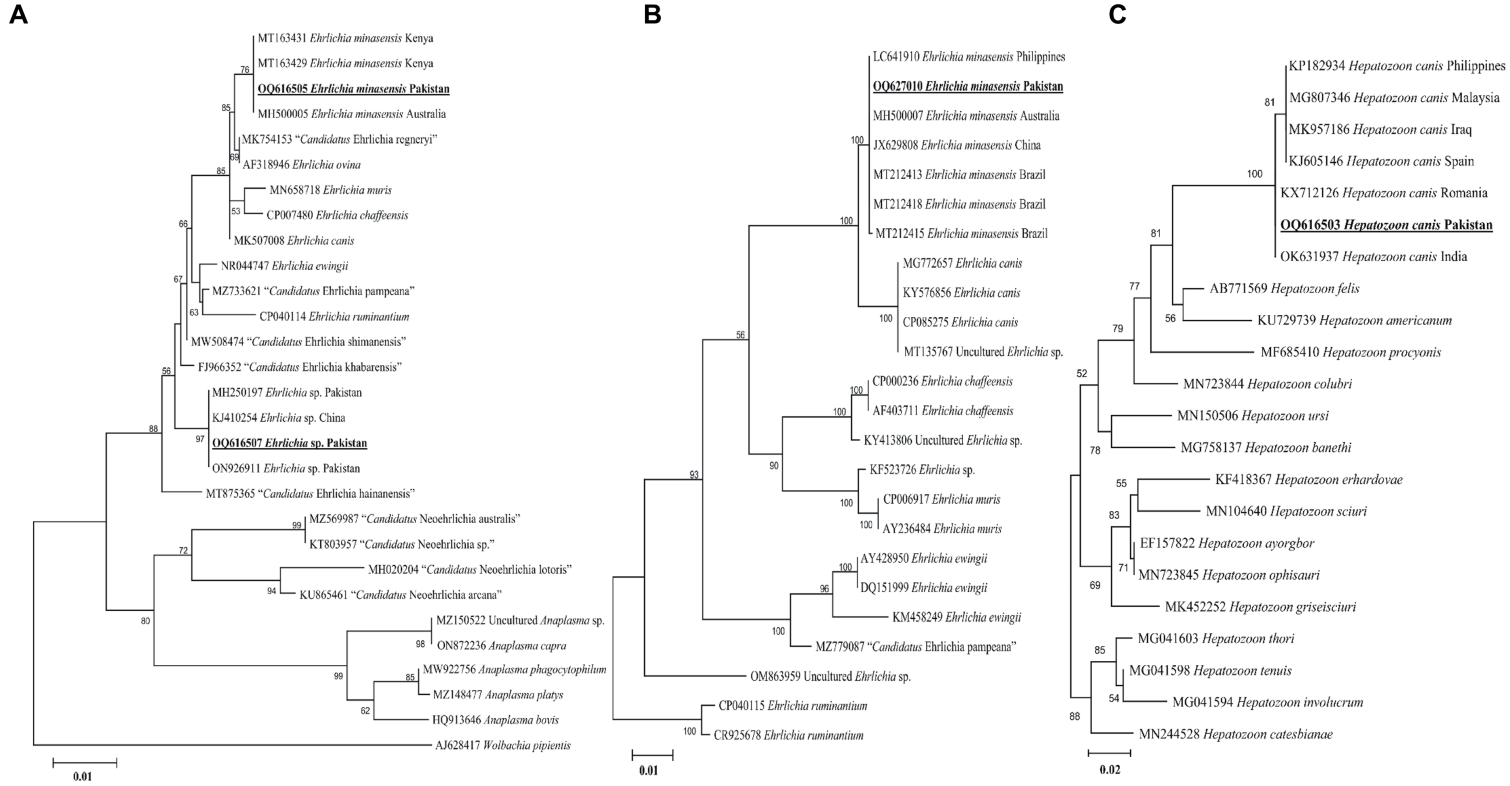
Maximum likelihood phylogenetic trees based on 16S rDNA **(A)** and *dsb* **(B)** for *Ehrlichia* spp. and 18S rDNA **(C)** partial sequences for *Hepatozoon canis*. The *Wolbachia pipientis* 16S rDNA, *Ehrlichia ruminantium dsb*, and *Hepatozoon catesbianae*, *Hepatozoon thori, Hepatozoon tenuis*, and *Hepatozoon involucrum* 18S rDNA sequences were used as an outgroup. The obtained sequences are shown in bold underline font.

According to the BLAST results, the 16S rDNA sequences of *Ehrlichia* sp. and *E. minasensis* showed 100% identity with the corresponding species. In the phylogenetic tree, the 16S rDNA sequences of *Ehrlichia* sp. and *E. minasensis* were clustered with the corresponding sequences (Figure 5A). In the case of the *dsb* sequence, the BLAST results of the *dsb* sequence of *Ehrlichia* sp. showed 100% identity with the *E. minasensis* sequence. In the phylogenetic tree, the *dsb* sequence of *E. minasensis* was clustered with the same species (Figure 5B). The obtained 16S rDNA and *dsb* sequences of *Ehrlichia* sp. and *E. minasensis* were deposited to the GenBank (Supplementary Table S2).

#### Hepatozoon canis

The obtained 18S rDNA sequence of *Hepatozoon* sp. showed 99–100% identity with the *H. canis* sequence. In the phylogenetic tree, the 18S rDNA sequence of *H. canis* was clustered with the same species (Figure 5C). The obtained 18S rDNA sequence of *H. canis* was deposited to the GenBank (Supplementary Table S2).

#### *Coxiella* spp.

**Figure 6:**
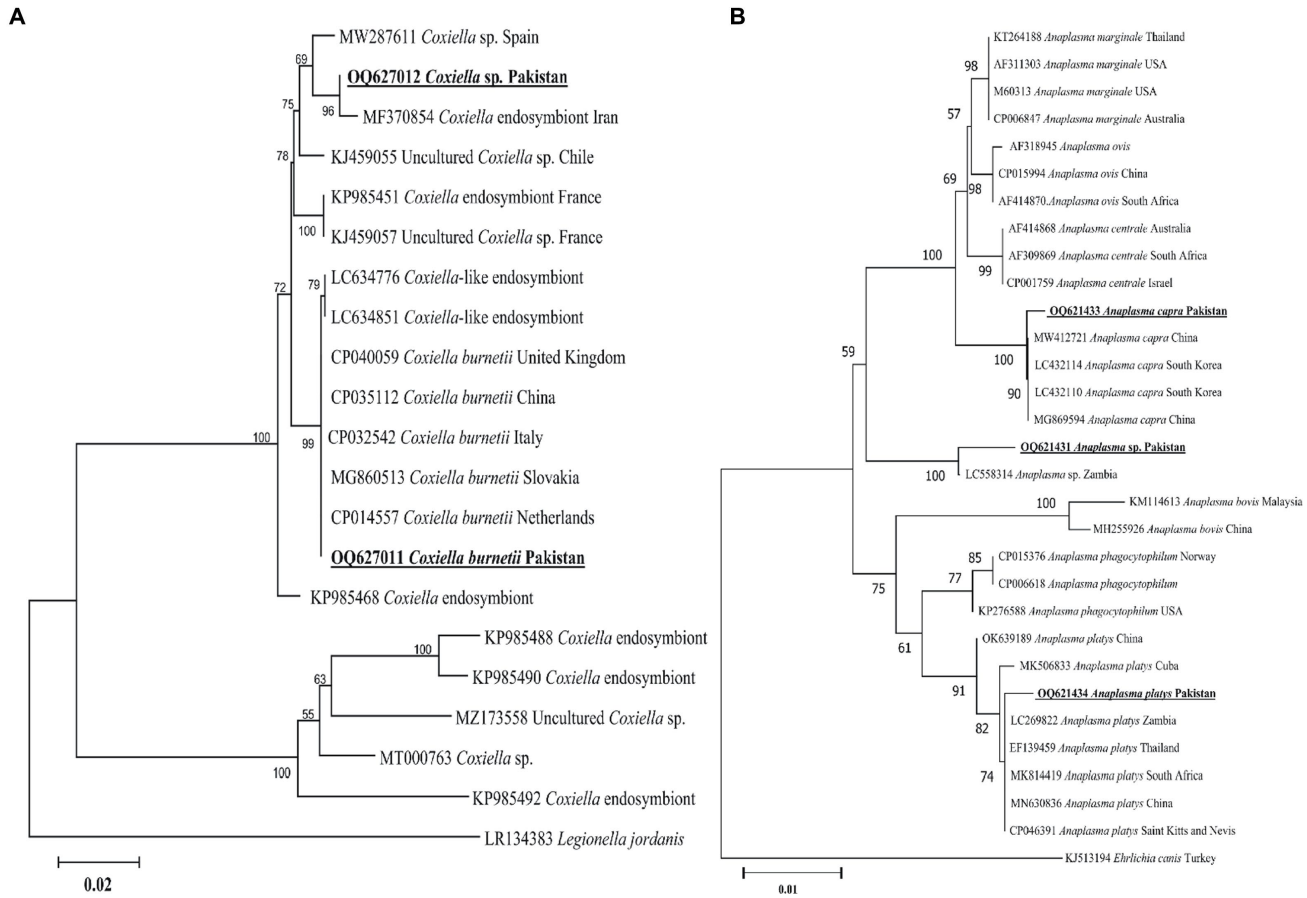
Maximum likelihood phylogenetic trees based on *groEL* **(A)** and 16S rDNA **(B)** partial sequences of *Coxiella* spp. and *Anaplasma* spp., respectively. The *Legionella jordanis groEL* and *Ehrlichia canis* 16S rDNA sequences were used as an outgroup. The obtained sequences are shown in bold underline font.

The present *groEL* sequence of *Coxiella* sp. showed 97–98% maximum identity with the *Coxiella* sp. sequence, followed by 96.55% identity with *C. burnetii*. Other *Coxiella* sp. *groEL* sequences showed 100% identity with *C. burnetii*. In the phylogenetic tree, the *groEL* sequence of *Coxiella* sp. was clustered with the *Coxiella* sp. endosymbiont sequences, while the sequence of *C. burnetii* was clustered with the corresponding species (Figure 6A). The obtained *groEL* sequences of *Coxiella* sp. and *C. burnetii* were deposited to the GenBank (Supplementary Table S2).

#### *Anaplasma* spp.

According to the BLAST results, 16S rDNA *Anaplasma* spp. sequences showed 99.12% maximum identity with *A. capra*, 100% maximum identity with *A. platys* sequences, and 99.13% maximum identity with an undermined *Anaplasma* sp. In the phylogenetic tree, the 16S rDNA sequences of *A. capra*, *A. platys*, and undetermined *Anaplasma* sp. were clustered with the corresponding species (Figure 6B). The obtained 16S rDNA sequences of *A. capra*, *A. platys*, and *Anaplasma* sp. were deposited to the GenBank (Supplementary Table S2).

#### *Rickettsia* spp.

**Figure 7:**
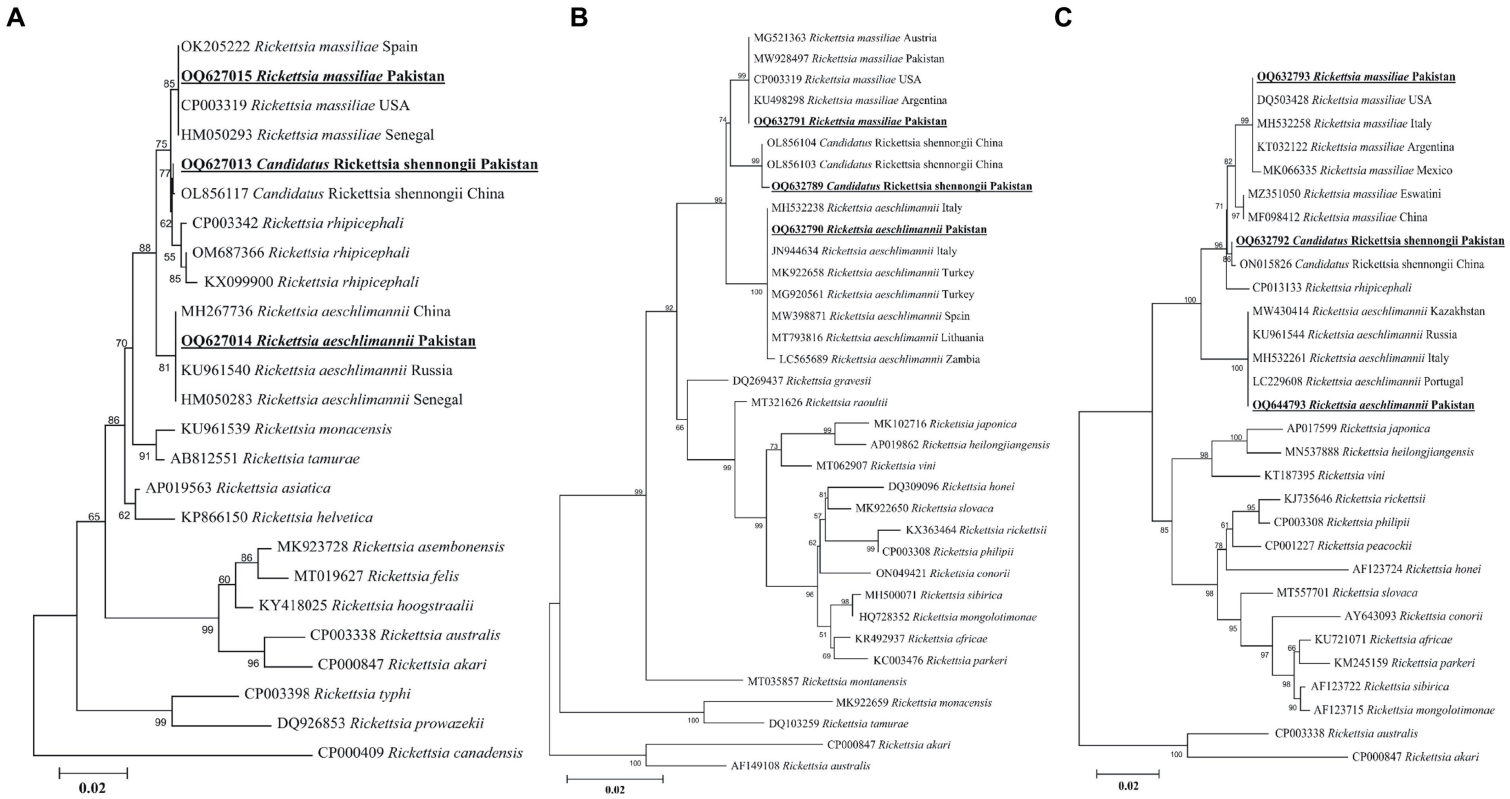
Maximum likelihood phylogenetic trees based on *gltA* **(A)**, *ompA* **(B)**, and ompB **(C)** partial sequences of *Rickettsia* spp. The *Rickettsia canadensis* gltA, *Rickettsia akari* and *Rickettsia australis* ompA, and *Rickettsia akari* and *Rickettsia australis* ompB sequences were used as an outgroup. The obtained sequences are shown in bold underline font.

A rickettsial *gltA* sequence showed 100% identity with *R. massiliae*, and in the phylogenetic tree, it was clustered with the corresponding species (Figure 7A). Another obtained *gltA* sequence showed 100% identity with “*Ca*. R. shennongii,” and it was phylogenetically clustered with the corresponding species (Figure 7A). A third rickettsial *gltA* sequence showed 100% identity with *R. aeschlimannii*, and in the phylogenetic tree, it was clustered with the corresponding species (Figure 7A).

The obtained *ompA* sequence showed 100% identity with *R. massiliae* and was phylogenetically clustered with the same species (Figure 7B). Another *ompA* sequence showed 100% identity with “*Ca*. R. shennongii” and in the phylogenetic tree, it was clustered with the same species (Figure 7B). A third *ompA* fragment showed 99.8–100% identity with *R. aeschlimannii* sequences, and in the phylogenetic tree, it was clustered with the same species (Figure 7B).

In the case of *ompB*, the obtained sequences showed 100% identity with *R. massiliae*. In the phylogenetic tree, the obtained *R. massiliae* sequence was clustered with the same species. Another *ompB* sequence showed 100% identity with “*Ca*. R. shennongii” and in the phylogenetic tree, this sequence was clustered with “*Ca*. R. shennongii” (Figure 7C). A third *ompB* sequence showed 100% identity with *R. aeschlimannii*, and it was phylogenetically clustered with the corresponding species (Figure 7C). The obtained *gltA*, *ompA*, and *ompB* sequences of *R. massiliae*, “*Ca*. R. shennongii” and *R. aeschlimannii* were deposited to the GenBank (Supplementary Table S2).

## Discussion

BASDs are commonly exposed to vector-borne pathogens due to the lack of adequate protection against ectoparasites in different regions. Different bacterial and protozoal infections have been reported in ticks infesting dogs, depending on the status of reservoir hosts, geographical variations in the exposure to tick vectors, detection methods, and the time of testing (2). The epidemiological situations of ticks and tick-borne pathogens associated with BASDs near butcher shops have not been previously investigated. A combination of morphological and molecular characterization resulted in the identification of several tick species infesting BASDs, including *Rh. sanguineus*, *Rh*. *haemaphysaloides*, *Rh. turanicus*, and *Rh. microplus*. The examined ticks were found positive for a variety of pathogenic and undetermined microorganisms, including species of the genera *Ehrlichia*, *Coxiella*, *Hepatozoon*, *Anaplasma*, and *Rickettsia*, which can be transmitted and pose health threats to humans, as well as wild and domestic animals. The different detected pathogenic and undermined species included *E. minasensis*, *Ehrlichia* sp., *H. canis*, *Coxiella* sp., *C. burnetii*, *A. capra*, *A. platys*, *Anaplasma* sp., *R. massiliae*, “*Ca*. R. shennongii” and *R. aeschlimannii*. This is the first investigation of *Ehrlichia* spp., *Hepatozoon* spp., *Coxiella* spp., *Anaplasma* spp., and *Rickettsia* spp. in ticks infesting BASDs. In Pakistan, *E. minasensis*, *H. canis*, *C. burnetii*, *A. capra*, *A. platys*, “*Ca*. R. shennongii” and *R. aeschlimannii* were detected for the first time, and they pose zoonotic threats to public health.

Abandoned stray dogs are frequently exposed and particularly vulnerable to the high risks of tick infestation and dispersal (5). Several tick species infest stray dogs in urban and peri-urban regions, and various pathogens are transmitted from dogs to other animals and humans (28). Surveillance requires a thorough understanding of tick dentification to reduce losses associated with tick-borne disease-causing agents (9, 16, 40). Morphological identification notwithstanding, the best way to determine the taxonomic status of the tick species is through molecular characterization utilizing 16S rDNA and *cox1* genetic markers (10, 15, 41, 42). The 16S rDNA and *cox1* sequences for *Rh. sanguineus*, *Rh. turanicus, Rh. microplus*, and *Rh. haemaphysaloides* in the present study revealed the closest identities of these ticks to the corresponding species from the Oriental and Palearctic regions.

BASDs freely roam outdoors, which increases their exposure to ticks and leads to uncontrolled breeding. They are mostly spotted in urban and rural localities. In general, stray dogs tend to carry a number of ticks, most commonly the dog tick *Rh. sanguineus*, which is a vector for several pathogens (43). Two *Ehrlichia* species (*E. minasensis* and *Ehrlichia* sp.) were detected in ticks in BASDs. In previous studies, *E. minasensis* has been detected in ticks infesting different hosts, such as cattle, horses, goats, and dogs (44, 45). For the genetic characterization of *Ehrlichia* spp., the 16S rDNA and *dsb* partial fragments have been identified as accurate genetic markers at the species level (46, 47). The 16S rDNA fragments of *Ehrlichia* spp. were closely related to the *Ehrlichia* sp. and *E. minasensis* species. In the 16S rDNA-based phylogeny, *Ehrlichia* sp. formed a sister clustered with *E. ewingii* and *E. ruminantium*, suggesting that this species may be of zoonotic concern. In the case of *dsb* fragments, the present *E. minasensis* sequences were phylogenetically clustered with the same species reported from the Palearctic, Neotropical, Australian, and Oriental regions. In both phylogenetic trees based on the 16S rDNA and *dsb*, the appearance of *E. minasensis* as a sister clade to *E. canis* might suggest that both are potentially canine pathogens (45, 47). Considering the detection of *Ehrlichia* spp. in ticks infesting free-roaming BASDs, further investigations are critical to disclose its association between stray dogs (BASDs) and humans.

*Hepatozoon canis* is a tick-borne apicomplexan parasite infecting canids and has long been known to be transmitted by ingesting infected *Rh. sanguineus* and *Rh. turanicus* ticks (48). The available phylogeny of *H. canis* is based on the 18S rDNA sequence, which has been demonstrated to be useful for inferring phylogenetic analyzes at the species level (48). Phylogenetic analysis based on the 18S rDNA sequence of this species suggests its close resemblance with the same species circulating in the Palearctic and Oriental regions.

More than 40 Ixodid (*Rhipicephalus, Dermacentor, Haemaphysalis*, *Amblyomma*, and *Ixodes*) and Argasid (*Ornithodoros* and *Argas*) tick species are known to harbor bacterial microbes related to *C. burnetii* and other *Coxiella-*like endosymbionts, which have been commonly detected in *Rhipicephalus* spp., particularly *Rh. sanguineus* (25, 49). *Rhipicephalus* spp. may play an important role in the transmission of *C. burnetii* among the hosts. Except for *C. burnetii*, there is no evidence that *Coxiella* spp. cause disease in the host; however, the pathogenic roles of these species have remained undetermined (49). Q fever is a vector-borne zoonotic disease caused by *C. burnetii* that affects a wide range of hosts globally. *Coxiella* spp. have been detected in *Rh. sanguineus* (s.l.) based on the *groEL* sequence (26). Since its successful detection in various ticks, the *groEL* sequences are potentially specific for *Coxiella*-like endosymbionts (18, 25). Likewise, the *groEL* partial sequence of *Coxiella* sp. and *C. burnetii* was detected in *Rhipicephalus* ticks of BASDs, which were closely related to the corresponding species.

Several tick species have been reported as vectors of *Anaplasma*, particularly in the genus *Rhipicephalus* (14, 50). Although *Anaplasma* spp. are prevalent in ixodid ticks, relatively limited studies have been conducted to investigate its detection, especially in BASD ticks (2). *Anaplasma* spp. were detected in high abundance in BASD ticks, which is nearly comparable to prior studies (2). *Anaplasma platys* and *A. capra* have mostly been considered to be transmitted by the dog tick *Rh. sanguineus*, and dogs have been confirmed as the hosts for *A. capra* in China (27). For the genetic characterization of *Anaplasma* spp., the highly conserved 16S rDNA marker has been historically employed (14, 27, 50). Likewise, the 16S rDNA fragments of *Anaplasma* spp., such as *A. capra* and *A. platys*, and an undermined *Anaplasma* sp. detected in *Rhipicephalus* spp. align with previous reports (14, 50). The detection of *Anaplasma* spp. in ticks infesting BASDs in Pakistan might suggest its geographic expansion, highlighting the need for further comprehensive studies on its pathogenicity to screen for the epidemiological and evolutionary status, which was previously underestimated due to the lack of sufficient data.

In Pakistan, earlier epidemiological investigations on the detection of *Rickettsia* spp. were mostly performed on ticks infesting small and large ruminants, equine, and wild hosts (11, 15, 16, 29, 51). Despite this gap, we used molecular screening of *Rickettsia* spp. in ticks infesting BASDs. Genetic markers, such as *gltA, ompA*, and *ompB*, have been utilized to determine a high degree of intraspecific variation and are extensively used for reliable characterization at the species level (42). We detected and characterized for the first time three rickettsial agents, *Rickettsia massiliae*, “*Ca*. Rickettsia shennongii” and *Rickettsia aeschlimannii* in ticks infesting BASDs. *Rickettsia massiliae* and *Rickettsia aeschlimannii* have been detected in ticks of multiple hosts in several districts of Pakistan (19, 52). The detection of “*Ca*. R. shennongii” represents the first record of any host or tick in Pakistan. Recently, this species was detected in *Rh. haemaphysaloides* ticks in China as a novel spotted fever group (SFGR) (25). The detection of “*Ca*. R. shennongii” in *Rh. sanguineus*, *Rh. haemaphysaloides*, and *Rh. turanicus* suggests its diverse host and distribution range and enforces the need to investigate its zoonotic threat to other hosts, including humans. These findings might advance our knowledge of the diversity of circulating tick-borne pathogens in the region, highlighting the need for further comprehensive surveillance studies to properly monitor ticks for potential zoonotic threats.

## Conclusion

To effectively manage ticks and tick-borne infections, the community must be educated regarding the associated risks and prevention strategies. The current endeavor highlights the risks of ticks infesting BASDs to public health. A comprehensive analysis was undertaken to determine which bacterial and protozoal pathogens might be carried by ticks infesting BASDs that inhabit butcher shops. This study will provide fundamental knowledge about the risks associated with these free-roaming BASDs, convincing health policymakers to ensure the control of any zoonotic consequences associated with these ticks infesting dogs near butcher shops. To minimize the risks of ticks and tick-borne diseases related to BASDs, proper disposal of meat and butcher waste should be adopted, and open-street butcher shops should be properly managed. Further surveillance studies are essential to understand the status of ticks and tick-borne pathogens in BASDs in different regions.

## Data availability statement

The datasets presented in this study can be found in online repositories. The names of the repository/repositories and accession number(s) can be found in the article/Supplementary material.

## Ethics statement

The animal studies were approved by the current study was approved by the Advance Studies Research Board (ASRB: Dir/A&R/AWKUM/2022/9396) committee members of the Faculty of Chemical and Life Sciences, Abdul Wali Khan University, Mardan Khyber Pakhtunkhwa (KP), Pakistan. Butchers were informed verbally about their observations and collection of ticks. The studies were conducted in accordance with the local legislation and institutional requirements. Written informed consent was obtained from the owners for the participation of their animals in this study.

## Author contributions

AiA designed the study. AiA, SU, and MN collected the ticks and carried out phylogenetic and statistical analysis. AiA, AdA, MA, and TT performed the experiments. All authors contributed to the article and approved the submitted version.

## Abbreviations

BASDs: Butcher-associated stray dogs
BLAST: Basic local alignment search too
*gltA*: Citrate synthase
SFGR: Spotted fever group *Rickettsia*
VBDs: Vector-borne diseases
KP: Khyber Pakhtunkhwa
MEGA: Molecular evolutionary genetics analysis
NCBI: National Center for Biotechnology Information
CME: Canine monocytic ehrlichiosis
Q fever: Query fever
*ompA*: Outer membrane protein
PCR: Polymerase chain reaction
rDNA: Ribosomal DNA
