# Supplementary material for: First report on tick-borne pathogens detected in ticks infesting stray dogs near butcher shops
Source: Front Vet Sci. 2023 Sep 18;10:1246871. doi: 10.3389/fvets.2023.1246871 (PMC10548827; doi:10.3389/fvets.2023.1246871)
Supplement: Supplementary file 1 [file Table_1.docx]

**Supplementary Table 1.** List of primers used for molecularly characterizing ticks and their associated tick-borne bacterial and protozoal species.

| **Organisms** | **Genes** | **Primers** | **Sequences, 5’-3’** | **Amplicons size** | **Annealing temperature** | **References** |
| --- | --- | --- | --- | --- | --- | --- |
| Ticks | 16S rDNA | 16S+1 | CCGGTCTGAACTCAGATCAAGT | 460 bp | 54°C | [53] |
|  |  | 16S-1 | GCTCAATGATTTTTTAAATTGCTGT |  |  |  |
| Ticks | *cox1* | cox1 F | GGAACAATATATTTAATTTTTGG | 850 bp | 55°C | [52] |
|  |  | cox1 R | ATCTATCCCTACTGTAAATATATG |  |  |  |
| *Anaplasma*/*Ehrlichia* spp. | 16S rDNA | EHR16SD | GGTACCYACAGAAGAAGTCC | 344 bp | 55°C | [54] |
|  |  | EHR16SR | TAGCACTCATCGTTTACAGC |  |  |  |
| *Anaplasma*/*Ehrlichia* spp. | *dsb* | DSB-330 F | GATGATGTTTGAAGATATSAAACAAAT | 349 bp | 52°C | [46] |
|  |  | DSB-380 F | ATTTTTAGRGATTTTCCAATACTTGG |  |  |  |
|  |  | DSB-720 R | CTATTTTACTTCTTAAAGTTGATAWATC |  |  |  |
| *Coxiella* spp. | *groEL****** | CoxGrF1 | TTTGAAAAYATGGGCGCKCAAATGGT | 619 bp | 56°C | [25] |
|  |  | CoxGrR2 | CGRTCRCCAAARCCAGGTGC |  |  |  |
|  |  | CoxGrF2 | GAAGTGGCTTCGCRTACWTCAGACG |  |  |  |
|  |  | CoxGrFR1 | CCAAARCCAGGTGCTTTYAC |  |  |  |
| *Hepatozoon* spp. | 18S rDNA | 18s HEP1F | GGTAATTCTAGAGCTAATACATGAGC | 574 bp | 50°C | [46] |
|  |  | 18s HEP4R | ACAATAAAGTAAAAAACAYTTCAAAG |  |  |  |
| *Rickettsia* spp. | *gltA* | CS‑78 | GCAAGTATCGGTGAGGATGTAAT | 401 bp | 48°C | [55] |
|  |  | CS‑323 | GCTTCCTAAAATTCAATAAATCAGGAT |  |  |  |
| *Rickettsia* spp. | *ompA* | Rr190.70 | ATGGCGAATATTTCTCCAAAA | 631 bp | 55°C | [56] |
|  |  | Rr190.701 | GTTCCGTTAATGGCAGCATCT |  |  |  |
| *Rickettsia* spp. | *ompB* | 120-M59 | CCGCAGGGTTGGTAACTGC | 862 bp | 50°C | [57] |
|  |  | 120-807 | CCTTTTAGATTACCGCCTAA |  |  |  |

*** Nested PCR**
