# Supplementary material for: First report on tick-borne pathogens detected in ticks infesting stray dogs near butcher shops
Source: Front Vet Sci. 2023 Sep 18;10:1246871. doi: 10.3389/fvets.2023.1246871 (PMC10548827; doi:10.3389/fvets.2023.1246871)
Supplement: Supplementary file 2 [file Table_2.docx]

**Supplementary Table 2.** List of accession numbers of the obtained sequences deposited to GenBank.

| **Accession number** | **Sequence size** | **Species name** | **Genetic marker** | **Cited figure** |
| --- | --- | --- | --- | --- |
| OQ600635 | 390 bp | *Rh. sanguineus* | 16S rDNA | Fig 3A |
| OQ600637 | 403 bp | *Rh. turanicus* | 16S rDNA | Fig 3A |
| OQ600636 | 396 bp | *Rh. microplus* | 16S rDNA | Fig 3A |
| OQ600638 | 385 bp | *Rh. haemaphysaloides* | 16S rDNA | Fig 3A |
| OQ621770 | 605 bp | *Rh. sanguineus* | *cox1* | Fig 3B |
| OQ621796 | 781 bp | *Rh. turanicus* | *cox1* | Fig 3B |
| OQ621750 | 793 bp | *Rh. microplus* | *cox1* | Fig 3B |
| OQ622004 | 600 bp | *Rh. haemaphysaloides* | *cox1* | Fig 3B |
| OQ616507 | 309 bp | *Ehrlichia* sp. | 16S rDNA | Fig 5A |
| OQ616505 | 331 bp | *E. minasensis* | 16S rDNA | Fig 5A |
| OQ627010 | 343 bp | *E. minasensis* | *Dsb* | Fig 5B |
| OQ616503 | 524 bp | *H. canis* | 18S rDNA | Fig 5C |
| OQ627012 | 551 bp | *Coxiella* sp. | *groEL* | Fig 6A |
| OQ627011 | 540 bp | *C.* *burnetii* | *groEL* | Fig 6A |
| OQ621433 | 342 bp | *A. capra* | 16S rDNA | Fig 6B |
| OQ621434 | 338 bp | *A. platys* | 16S rDNA | Fig 6B |
| OQ621431 | 346 bp | *Anaplasma* sp. | 16S rDNA | Fig 6B |
| OQ627015 | 348 bp | *R. massiliae* | *gltA* | Fig 7A |
| OQ627013 | 348 bp | ‘*Ca.* R. shennongii’ | *gltA* | Fig 7A |
| OQ627014 | 354 bp | *R. aeschlimannii* | *gltA* | Fig 7A |
| OQ632791 | 564 bp | *R. massiliae* | *ompA* | Fig 7B |
| OQ632789 | 564 bp | ‘*Ca.* R. shennongii’ | *ompA* | Fig 7B |
| OQ632790 | 488 bp | *R. aeschlimannii* | *ompA* | Fig 7B |
| OQ632793 | 735 bp | *R. massiliae* | *ompB* | Fig 7C |
| OQ632792 | 774 bp | ‘*Ca.* R. shennongii’ | *ompB* | Fig 7C |
| OQ644793 | 773 bp | *R. aeschlimannii* | *ompB* | Fig 7C |
